# Method for User Interface of Large Displays Using Arm Pointing and Finger Counting Gesture Recognition

**DOI:** 10.1155/2014/683045

**Published:** 2014-09-01

**Authors:** Hansol Kim, Yoonkyung Kim, Eui Chul Lee

**Affiliations:** Department of Computer Science, Sangmyung University, Seoul 110-743, Republic of Korea

## Abstract

Although many three-dimensional pointing gesture recognition methods have been proposed, the problem of self-occlusion has not been considered. Furthermore, because almost all pointing gesture recognition methods use a wide-angle camera, additional sensors or cameras are required to concurrently perform finger gesture recognition. In this paper, we propose a method for performing both pointing gesture and finger gesture recognition for large display environments, using a single Kinect device and a skeleton tracking model. By considering self-occlusion, a compensation technique can be performed on the user's detected shoulder position when a hand occludes the shoulder. In addition, we propose a technique to facilitate finger counting gesture recognition, based on the depth image of the hand position. In this technique, the depth image is extracted from the end of the pointing vector. By using exception handling for self-occlusions, experimental results indicate that the pointing accuracy of a specific reference position was significantly improved. The average root mean square error was approximately 13 pixels for a 1920 × 1080 pixels screen resolution. Moreover, the finger counting gesture recognition accuracy was 98.3%.

## 1. Introduction

A significant amount of research has been conducted on hand gesture recognition. To perform interactive navigation and manipulation, pointing gesture and finger gesture recognition should be simultaneously executed.

Pointing gesture recognition methods can be categorized into two method types: two-dimensional (2D) image-based methods and three-dimensional (3D) methods. Although 2D image-based methods, dating back several decades, can be easily implemented today, their targeting accuracies are poor in comparison to more recent 3D methods. Therefore, 2D image-based methods are not considered in this paper.

Since the development of low cost, high depth perception 3D cameras, such as the Bumblebee and Kinect, 3D-based pointing gesture recognition methods have been widely researched. Yamamoto et al. proposed a real-time arm pointing gesture recognition method using multiple stereo cameras [1]. Because multiple stereo cameras cover a relatively wide area, the degree of freedom of a user's movement is relatively high. However, the calibration required to define epipolar geometric relations among multiple stereo cameras is considerably expensive. Other methods [2, 3] have considered head orientation to accurately estimate the hand pointing position. Head orientation typically changes as the hand targeting position changes. However, head orientation data cannot be reliably obtained, which can degrade the accuracy of the estimated hand targeting position. Another method [4] approached this problem by analyzing appearance, interactive context, and environment. However, the individual variations of these additional parameters can also lead to decreased targeting accuracy.

Recently, pointing gesture methods based on the skeleton model of the Kinect SDK (Software Development Kit) have been reported [5]. One particular pointing gesture method that was proposed utilized the skeleton model and a virtual screen [6]. The critical issue in this method, however, was defining a correspondence between the virtual screen and the physical display. In addition, this method did not consider self-occlusion;, it did not specifically address the issue of distinguishing both hand and shoulder points on a perspective line. Other 3D-based methods [7, 8] have also failed to address this issue. Although 3D-based methods are accurate in terms of defining a pointing vector for a fingertip, unstable dithering problems caused by low-resolution images can occur when a camera is positioned at a distance [9].

To facilitate interactive display manipulation, many finger gesture recognition methods have been studied. In a previous research effort [11], a fingertip detection method that combined depth images with color images was proposed. In this method, a finger outline tracking scheme was used, and its accuracy was relatively high. However, because the operational distance between the camera and hand was relatively short, the method cannot be considered in our large display and long distance environment. An appearance-based hand gesture recognition method using PCA (Principal Component Analysis) was described [12]. However, this method presents problems such as illuminative variation and hand orientation, which are similar to problems observed in PCA-based face recognition. In an alternative approach, a 3D template-based hand pose recognition method was proposed [13]. In this method, a 2D hand pose image was recognized by comparing 26 DOF (Degree of Freedom) 3D hand pose templates. However, the method is tightly coupled with a predefined 3D hand pose template. In addition, the computational complexity for estimating 3D hand poses from the captured 2D image stream was high. In a current research, a new hand posture recognition method was proposed based on the sparse representation of multiple features such as gray-level, texture, and shape [14]. However, this method is strongly dependent on a training database. Furthermore, the binary decision for each feature's sparsity presents a problem, because continuous values of sparse features must be considered.

To solve the problems related to previous pointing and hand gesture methods, a new arm pointing and finger counting gesture recognition method is proposed in this paper. Our proposed method is a user-dependent, calibration-free method based on the Kinect skeleton model. We resolve the self-occlusion problem in the arm pointing gesture recognition module. Moreover, finger counting gesture recognition is accurately performed using a low-resolution depth image. Both gesture recognition techniques are performed with a single Kinect device.

## 2. Proposed Method

**Figure 1:**
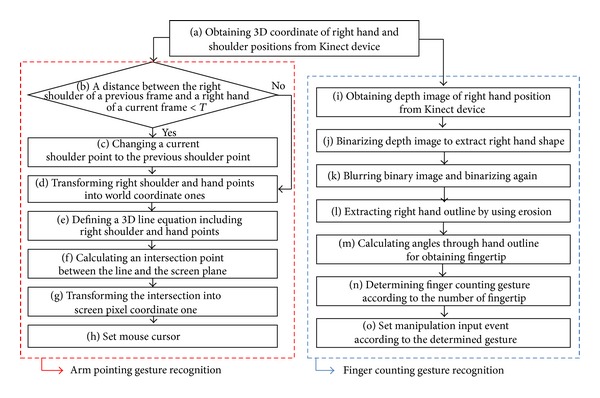
Flow diagram of the proposed method.

Our proposed method is executed as per the steps shown in Figure 1. The method is organized into two parts, namely, arm pointing gesture recognition and finger counting gesture recognition.

### 2.1. Arm Pointing Gesture Recognition

**Figure 2:**
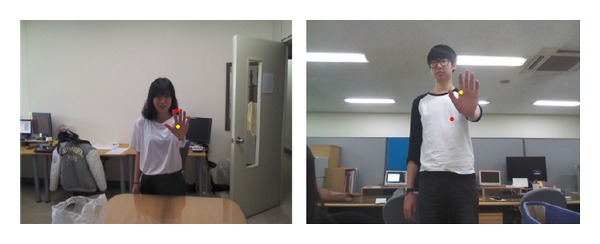
Examples of shoulder point detection errors caused by the self-occlusion problem (red dot: shoulder point, yellow dot: hand point).

Arm pointing gesture recognition is performed using the sequence shown in the red dotted box of Figure 1. First, 3D coordinates of the right-hand and shoulder positions are obtained using the skeleton model of the Kinect SDK. In the visible image captured from the Kinect device, the *X* and *Y* values of an arbitrary pixel's 3D coordinates are the same as their corresponding pixel coordinates in the visible image. The *Z* value, which is measured by the Kinect's depth camera, is multiplied by 10 mm. Next, we proceed to step (b), in which the Euclidean distance between the shoulder position in the previous frame and the hand position in the current frame is measured. When both the hand and shoulder positions lie on the same camera perspective line, the shoulder position cannot be accurately detected because of occlusion by the hand, as shown in Figure 2. We use exception handling to address such self-occlusion; if the distance measured in step (b) of Figure 1, specifically, is less than the empirically defined threshold (*T* = 10 pixels), the current shoulder position is set to that of the previous frame (step (c)). If the distance is greater than *T*, exception handling is not performed (i.e., step (c) is bypassed).

**Figure 3:**
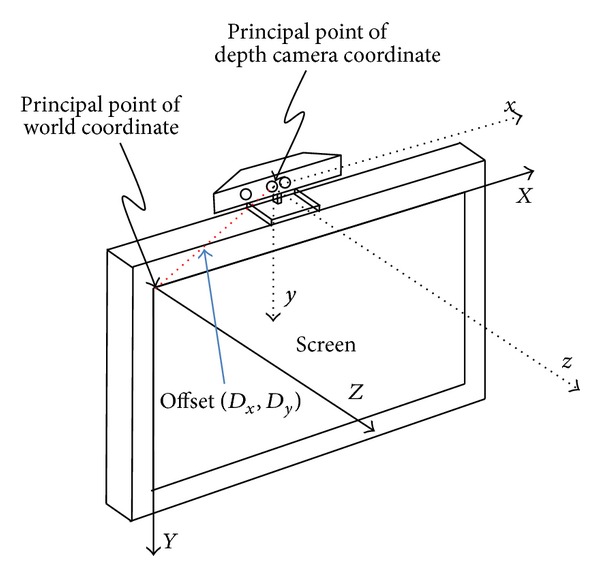
Geometric relation between the world coordinates and depth camera coordinates [10].

In the following step, the hand and potentially compensated shoulder coordinates (based on threshold *T*) are transformed into world coordinates. As shown in Figure 3, the principal point of the world coordinates is located in the top-left position of the screen. The transformation is performed according to the following equations [15]:

(1) $$\begin{array}{c} x_{k}=\left(i-\frac{w}{2}\right)\times \left(z_{k}+\text{minDist}\right)\times \text{SF}\times \frac{w}{h}\;, \end{array}$$

(2) $$\begin{array}{c} y_{k}=\left(j-\frac{h}{2}\right)\times \left(z_{k}+\text{minDist}\right)\times \text{SF}, \end{array}$$

(3) $$\begin{array}{c} z=z_{k}+4000, \end{array}$$

where minDist = − 10 and SF =0.0021 are based on the calibration results of previous works [11] and *i*and *j*are the horizontal and vertical pixel positions of the captured image frame with a spatial resolution of 640 × 480 pixels. Because the default *Z*-distance value (*z*_*k*_) can be as small as 400 mm, the *Z*-axis value in (3) must be compensated accordingly. Moreover, because the 3D coordinates (*x*_*k*_, *y*_*k*_, *z*) are measured from the principal point of the depth camera, the values of* x*_*k*_ and *y*_*k*_ should be adjusted by the offset ((*D*_*x*_, *D*_*y*_) in Figure 3) between the two principal world coordinate points and the depth camera coordinates. In our system configuration, *D*_*x*_ and *D*_*y*_ were 4450 mm and 950 mm, respectively, and were measured manually. The orientation variation between the Kinect and the screen is ignored. That is,

(4) $$\begin{array}{c} x=x_{k}+D_{x},\;\;y=y_{k}-D_{y}.\; \end{array}$$

The two world coordinate positions for the shoulder and hand are given by (*X*_*s*_, *Y*_*s*_, *Z*_*s*_) and (*X*_*e*_, *Y*_*e*_, *Z*_*e*_), respectively. Next (step (e) in Figure 1), a 3D line equation is defined from these two 3D points using the following equation:

(5) $$\begin{array}{c} \frac{X_{s}-X}{X_{s}-X_{e}}=\frac{Y_{s}-Y}{Y_{s}-Y_{e}}=\frac{Z_{s}-Z}{Z_{s}-Z_{e}}. \end{array}$$

Because the line equation is regarded as an arm-pointing vector and the planar equation of the screen is *z* = 0, the intersection point (*X*_*i*_, *Y*_*i*_) between the screen and the line equation is calculated in step (f) in Figure 1 as follows:

(6) Xi=−Ze(Xs−Xe)Zs−Ze+Xe,Yi=−Ze(Ys−Ye)Zs−Ze+Ye.

**Figure 4:**
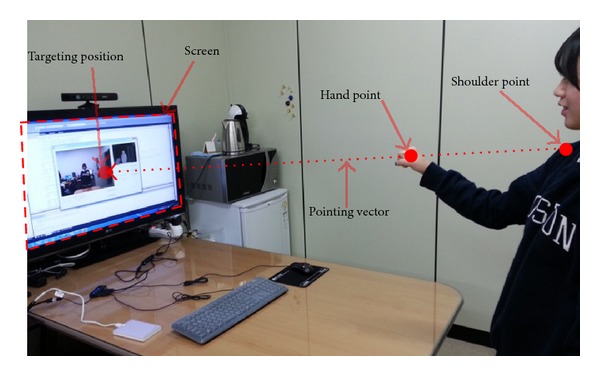
Conceptual diagram for representing the pointing vector and targeting position [10].

The intersection point is the physical targeting position shown in Figure 4. Because the physical targeting position (*X*_*i*_, *Y*_*i*_) is given in millimeters, its position must be transformed into logical pixel coordinates (*x*_*p*_, *y*_*p*_) in order to control the system mouse cursor position (step (g) of Figure 1). These logical pixel coordinates are given by

(7) $$\begin{array}{c} x_{p}=\frac{X_{i}\cdot x_{\text{res}}}{W},\;\;y_{p}=\frac{Y_{i}\cdot y_{\text{res}}}{H}, \end{array}$$

where (*x*_res_, *y*_res_) is the spatial resolution of the screen and *W* and *H* are the actual width and height of the screen, respectively. For our system, (*x*_res_, *y*_res_) = (1920, 1080), *W* = 932 mm, and *H* = 525 mm. Finally, the cursor position of the system mouse is moved to the calculated arm pointing position (*x*_*p*_, *y*_*p*_) using the WINAPI function SetCursorPos(int *x*, int *y*) [16].

### 2.2. Finger Counting Gesture Recognition

**Figure 5:**
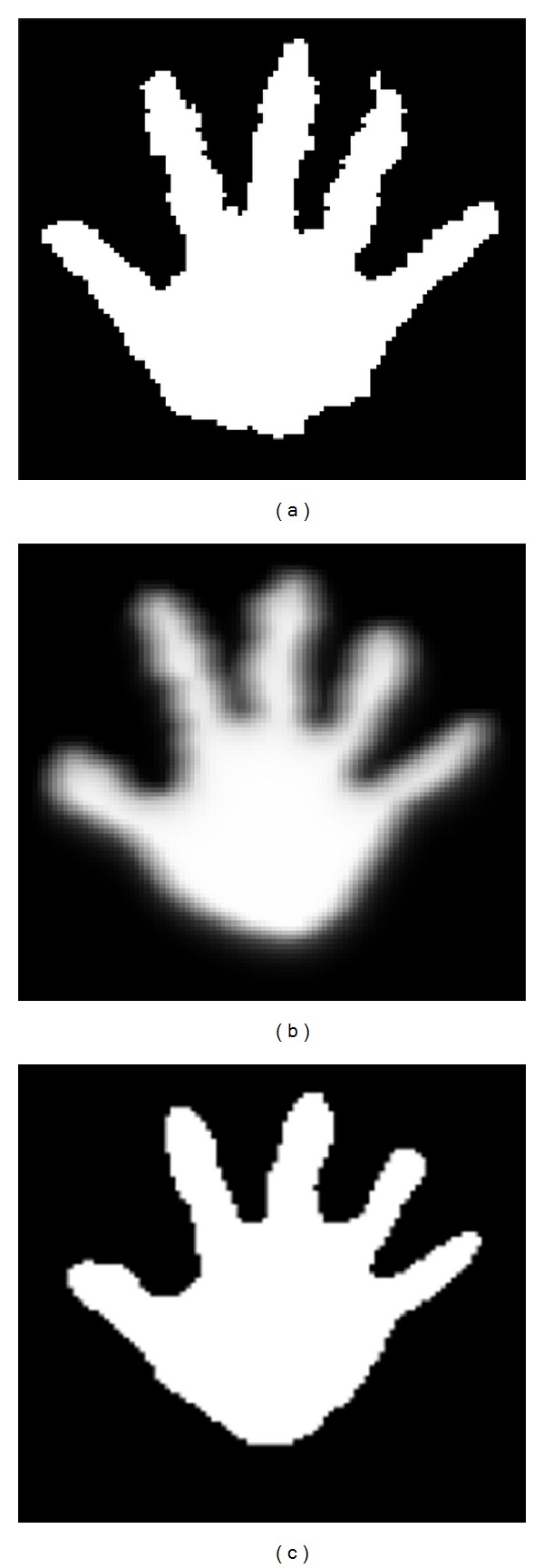
Binarized right hand images; (a) once binarized image, (b) blurred image with 7 ∗ 7 average filter, and (c) twice binarized image.

Finger counting gesture recognition is processed using the steps in the blue dotted box of Figure 1. In step (i), the right hand depth image is obtained based on the position of the right hand, which is acquired by using the Kinect SDK skeleton model. The spatial resolution of the image is 100 × 100. The gray levels of the depth image indicate the *Z*-distance between the Kinect depth camera lens and the corresponding object. Therefore, the higher the gray level, the shorter the distance between the camera lens and the object. In order to extract the right hand's shape, the right hand depth image is binarized by regarding the higher gray level in the depth image as the threshold (step (j) in Figure 1). However, an outline of right hand shape that has been binarized only once will be articulated, as shown in Figure 5(a). An extracted edge from a once-binarized right hand image will contain bifurcation, which may disturb fingertip detection that uses edge tracking. To solve this problem, a once-binarized right hand image is blurred by using a 7 × 7 average filter, as shown in Figure 5(b). Subsequently, a binarization is performed again using the median gray value (128 in a 0–255 gray scale) to obtain a right- hand shape (step (k) in Figure 1). A hand shape image with a flattened outline can be acquired, as shown in Figure 5(c).

Then, hand outline detection must be performed, to facilitate fingertip detection. Assuming that the twice-binarized image and the structural element for morphological erosion (⊙) are *A* and *B*, respectively, the hand outline image (*β*(*A*)) can be extracted by subtracting the erosion image from *A* (step (l) in Figure 1) using the following equation:

(8) $$\begin{array}{c} \beta \left(A\right)=A-\left(A⊙B\right). \end{array}$$

**Figure 6:**
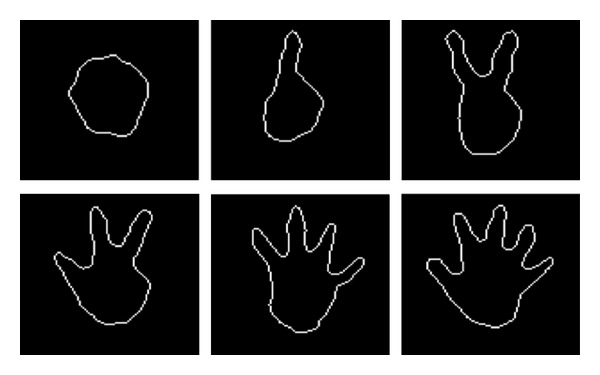
Examples of right hand outline images obtained by the proposed method.

As a result, the outline image of the right hand can be acquired as shown in Figure 6.

**Figure 7:**
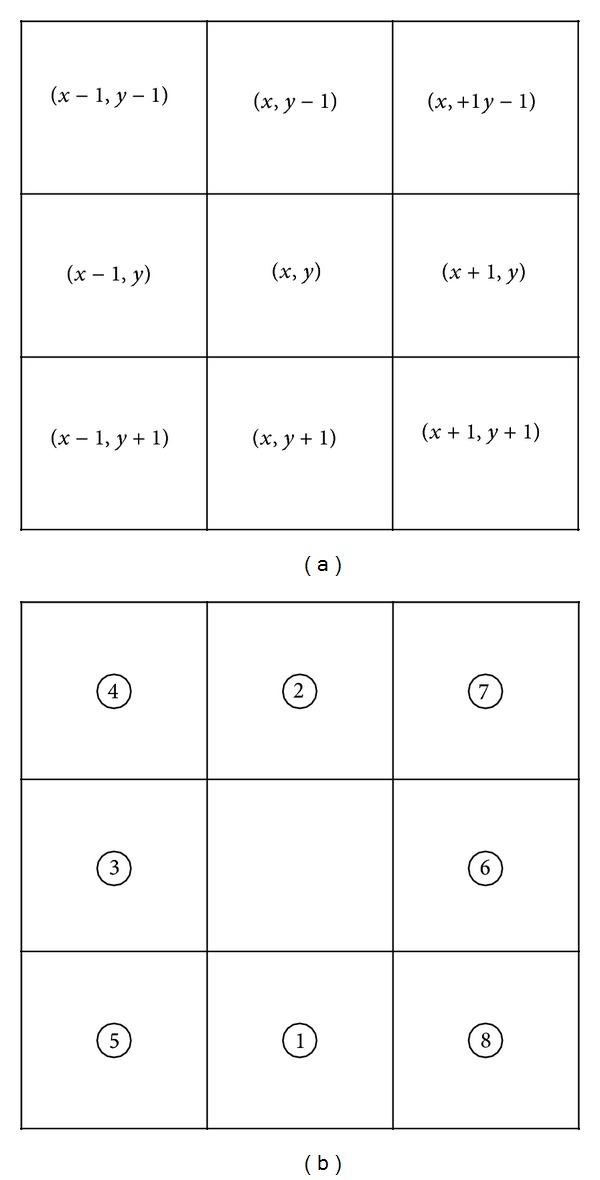
(a) 8-neighbor pixels and (b) assigned priority of the 8-neighbor pixels.

Subsequently, counterclockwise edge tracking is performed; the edge pixel that has the minimum *Y*-axis value is used as the starting point. If two points on the edge have the same minimum *Y*-axis value, the point with the lowest *X*-axis value is used as the starting point. The 8-neighbor pixels (Figure 7(a)) surrounding the starting point are assigned priorities 1 through 8, as shown in Figure 7(b).

According to the priority, the 8-neighbor pixels are analyzed to determine whether the pixel is an edge (gray level value = 255) and whether it is “nonvisited.” If an edge pixel that is “nonvisited” is detected among the 8-neighbor pixels, the pixel is determined to be the new center position. Accordingly, the previous center position is marked as “visited.” These steps are repeated until no pixels are found that satisfy the two conditions (edge and nonvisited) among the 8-neighbor pixels.

**Figure 8:**
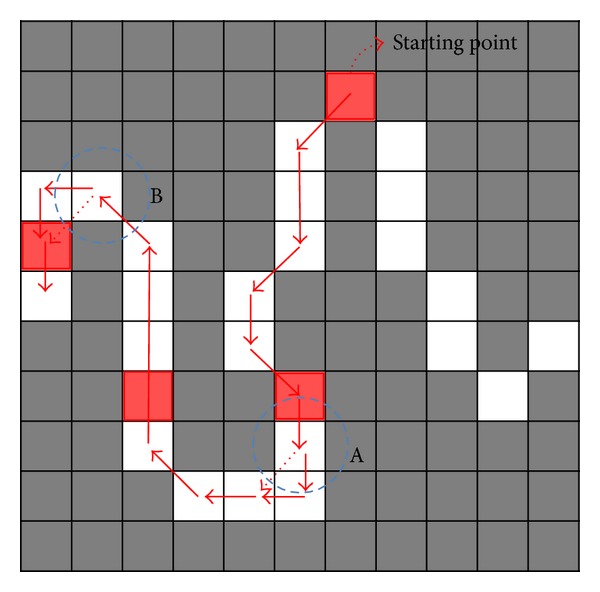
Edge tracking path example for explaining the necessity of the predefined priority (full arrow: normal edge tracking process, dotted arrow: abnormal edge tracking process).

If an 8-neighbor pixel priority is not assigned, edge tracking will be performed abnormally. For example, in the right hand edge of Figure 8, the minimum *Y*-axis value is determined as the starting point and is labeled in the figure. Edge tracking is performed by using the starting point as a center position. Then, the (*x* − 1, *y* + 1) pixel of the starting point's 8-neighbor pixels is changed to the next center point, according to the predefined priority order. If the (*x* + 1, *y* + 1) pixel has a higher priority than the (*x* − 1, *y* + 1) pixel, the priority is appropriate for clockwise edge tracking. Therefore, 8-neighbor pixels that have a value of (*x* − 1) as their *X*-index are assigned a higher priority than pixels that have (*x* + 1) as their *X*-index, to facilitate counterclockwise edge tracking. Edge tracking proceeds normally until arriving at position A. In position A, if the (*x* − 1, *y* + 1) pixel of the center point has a higher priority than the (*x*, *y* + 1) pixel, the (*x*, *y* + 1) pixel will not be visited. Then, in case the priority of the (*x* + 1, *y*) pixel is higher than the (*x* − 1, *y*) pixel, edge tracking will terminate abnormally when the bottom of A becomes the center position. Likewise, in position B, if the (*x* − 1, *y* − 1) pixel has a higher priority than the (*x* − 1, *y*) pixel, the (*x* − 1, *y*) pixel will not be visited and edge tracking will terminate abnormally. To prevent these abnormal cases, edge tracking should be performed according to a predefined priority.

**Figure 9:**
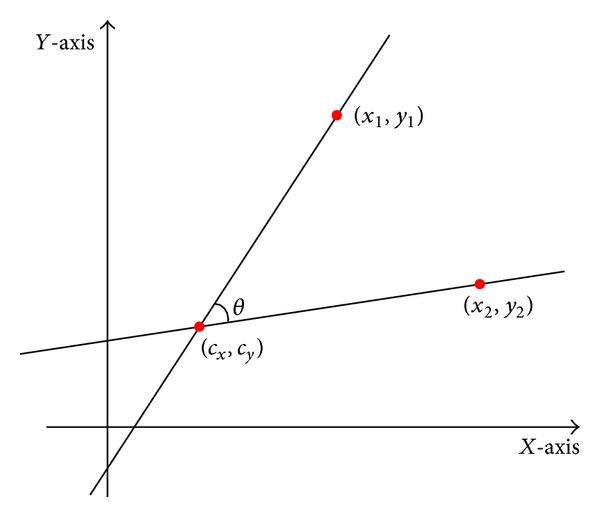
Example with three arbitrary points in a 2D coordinate.

While edge tracking is performed, three sequential points, at fifth-next-adjacent intervals, must be extracted as shown in Figure 8 (red points). Then, the angle between the three extracted points as Figure 9 must be calculated, using the following equation (step (m) in Figure 1):

(9) $$\begin{array}{c} \theta =\left(\tan^{-1}\left(\frac{y_{1}-c_{y}}{x_{1}-c_{x}}\right)-\;\tan^{-1}\left(\frac{y_{2}-c_{y}}{x_{2}-c_{x}}\right)\right)*\frac{180}{\pi }. \end{array}$$

**Figure 10:**
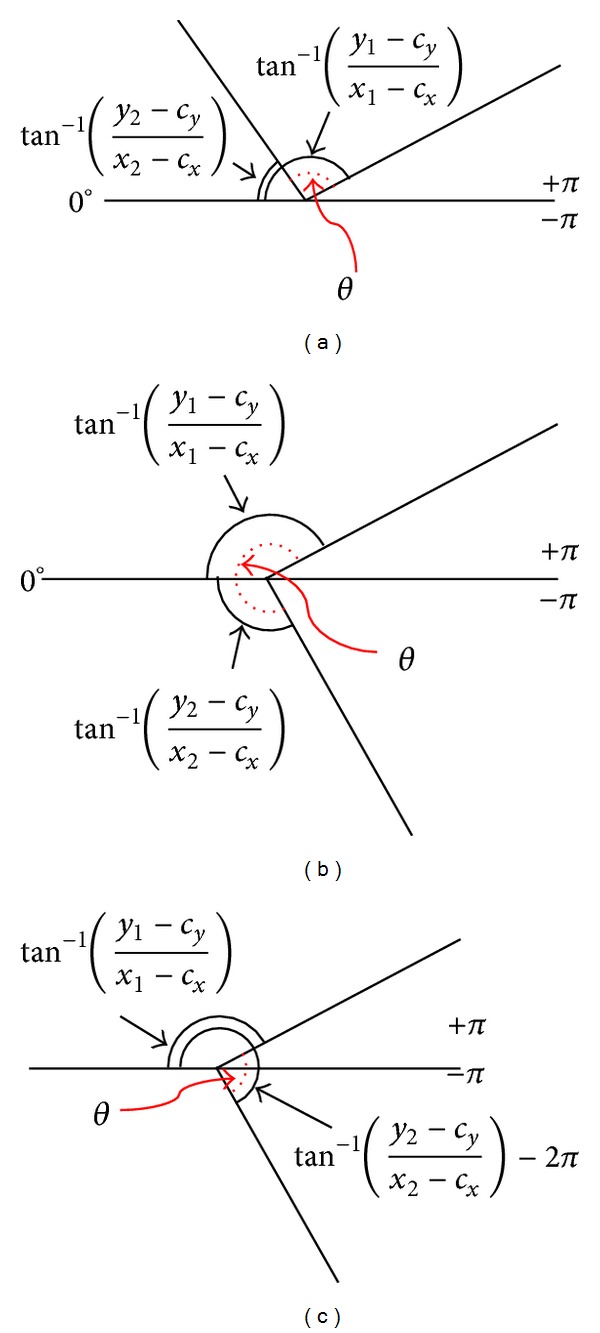
Angle calculation using three sequential points of the hand's outline edge. (a) Obtaining angle in a normal case. (b) Obtaining the opposite angle in error case. (c) Compensating error case (*θ*: calculated angle of three points at the intervals of five adjacent edge points).

Here, the angle of the three points is calculated using the atan2 function included in math.h header of the C standard library [17]. However, the atan2 function's output ranges are −*π* to *π*. Therefore, if the value of tan^−1^⁡((*y*_2_ − *c*_*y*_)/(*x*_2_ − *c*_*x*_)) is negative and the value of tan^−1^⁡((*y*_1_ − *c*_*y*_)/(*x*_1_ − *c*_*x*_)) is positive, the opposite angle of the three points will be calculated, as shown in Figure 10(b).

To solve this problem, the angle of the three points is calculated using the following equation, as illustrated in Figure 10(c):

(10) $$\begin{array}{c} \theta =\left(\left(\tan^{-1}\left(\frac{y_{2}-c_{y}}{x_{2}-c_{x}}\right)-2\pi \right)-\tan^{-1}\left(\frac{y_{1}-c_{y}}{x_{1}-c_{x}}\right)\right)*\frac{180}{\pi }.\; \end{array}$$

Then, if *θ* is lower than the predefined threshold (*T* = 110°), the center point of the three points is regarded as the fingertip (steps (n) and (o) in Figure 1). Finally, exception handling will be performed if one of the two noncenter points has already been identified as a fingertip, because if two of the three extracted points satisfy the condition, this indicates that the two points are on the same fingertip.

## 3. Experimental Result

**Figure 11:**
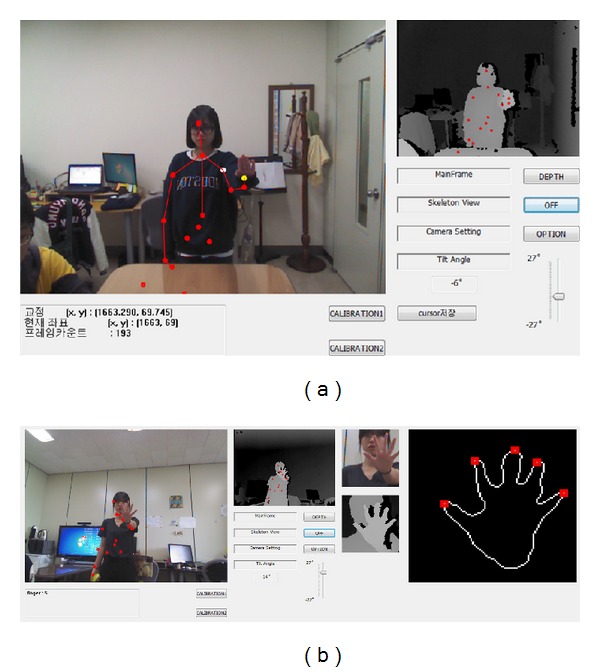
Lab-made gesture recognition software based on the upper body skeleton model in the Kinect SDK. (a) Pointing gesture recognition software (white dot: shoulder point, yellow dot: hand point) [10]. (b) Finger counting gesture recognition software.

To validate the proposed method, experiments were performed to measure the accuracy of the arm pointing and finger counting gesture recognition techniques. In the experiments, the distance between the subject's body and the screen was approximately 2.2 m. Software capable of recognizing upper body pointing gestures was implemented using C++, MFC (Microsoft Foundation Classes), and the Kinect SDK. The implemented software, as shown in Figure 11, could be operated in real time (approximately 18.5 frames/s) without frame delay or skipping on a PC with an Intel i7-3770 CPU, 8 GB RAM, and a 42-inch display.

**Figure 12:**
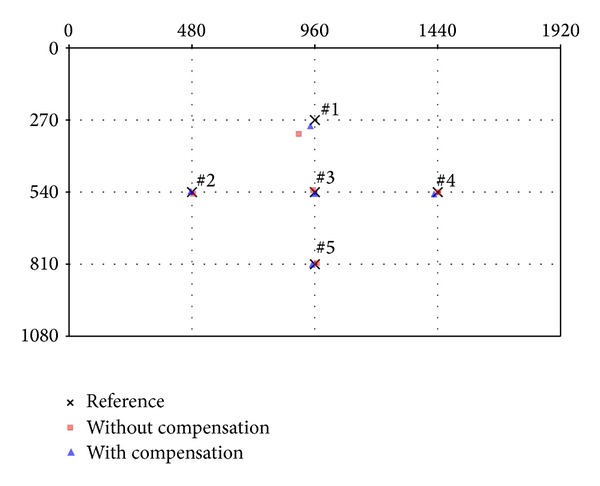
Experimental results of detecting target positions against reference positions [10].

In our first experiment, targeting accuracy for specific pointing positions was measured for eight subjects. Each subject pointed to five predefined reference positions (indicated by the “×” in Figure 12); this sequence was repeated three times. The indicated order was assigned randomly. Tests were performed with and without the self-occlusion compensation function in order to validate the performance of our proposed compensation method.

The measured accuracy results from the experiment are shown in Figure 12 and Table 1. Four outliers caused by detection errors of the hand or shoulder were not included. As shown in Figure 12 and Table 1, position 1 experienced a much higher error rate compared to the other reference positions. This can be attributed to self-occlusion occurring most frequently in position 1; specifically, both 3D shoulder and hand points are positioned on a single camera perspective line. After adopting the proposed compensation method, we confirmed improvements in targeting accuracy for position 1. In this case, the* X*-axis error received more compensation than that of the* Y*-axis, as shown in Table 1. The average RMS errors from tests with and without self-occlusion compensation were approximately 21.91 pixels and 13.03 pixels, respectively.

**Figure 13:**
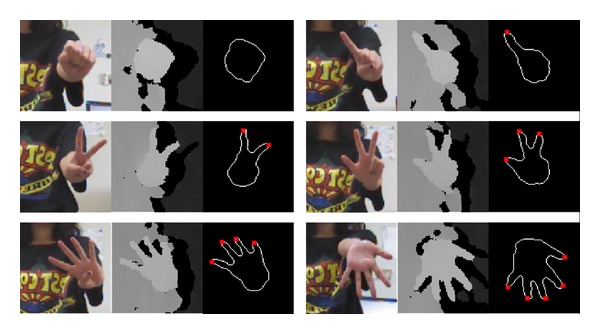
Examples of six different finger gestures used in the second experiment.

In our second experiment, the accuracy of the finger counting gesture recognition method was evaluated to validate the fingertip detection method. Five subjects participated in the experiment. Each subject performed six predefined finger-counting gestures, regardless of hand orientation, as shown in Figure 13. The order of the finger gestures was randomly announced. The accuracy was measured by comparing the number of fingers in the hand gesture to the number of fingertips that were detected.

**Figure 14:**
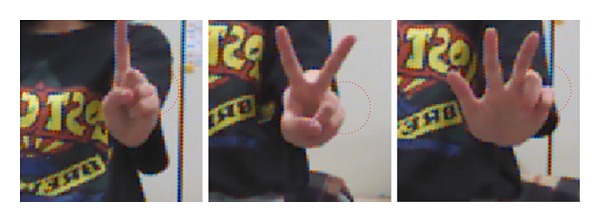
Shape comparison of folded ring and little fingers for one-, two-, and three-finger gestures.

Experimental results from the accuracy measurement are listed in Table 2. Here, the accuracy of the three-finger gesture was lower, compared to the other finger counting gestures. As shown in Figure 14, the shape of the folded ring and little fingers in the three-finger gesture is sharper than that in the one- and two-finger gestures. In one- and two-finger gestures, the thumb suppresses the folded ring and little fingers. Because the sharper shape of the ring and little finger in the three-finger gesture can be misinterpreted as fingertips, the three-finger gesture may have been interpreted as a four- or five-finger gesture. As a result, the average fingertip recognition accuracy for the six predefined finger gestures was 98.3%.

As shown in Table 3, the processing times for arm pointing and finger counting gesture recognition were considerably fast: 6.1 ms and 0.5 ms, respectively. The skeleton model detection time was not included in the calculated times. These experiments demonstrate that our proposed method can accurately recognize pointing and counting gestures in an efficient manner.

## 4. Conclusion

In this paper, we proposed a method for performing both pointing gesture and finger gesture recognition for large display environments, using a single Kinect device and a skeleton tracking model. To prevent self-occlusion, a compensation technique was designed to correct the shoulder position in cases of hand occlusion. In addition, finger counting gesture recognition was implemented based on the hand position depth image extracted from the end of the pointing vector. Experimental results showed that the pointing accuracy of a specific reference position significantly improved by adopting exception handling for self-occlusions. The average root mean square error was approximately 13 pixels for a 1920 × 1080 pixels screen resolution. Furthermore, the accuracy of finger counting gesture recognition was 98.3%.

In future works, we will define effective manipulation commands for the detected finger counting gestures. Further, the proposed method will be applied to immersive virtual reality contents [18–20] as a natural user interface method for performing interactive navigation and manipulation.

## Figures and Tables

**Table 1:** Targeting error against reference positions [10].

Reference positions	Error without compensation			Error with compensation		
	*X*-axis	*Y*-axis	RMS	*X*-axis	*Y*-axis	RMS
1	62.90	52.19	81.73	16.95	22.04	27.81
2	4.54	5.95	7.49	4.29	3.41	5.48
3	6.33	6.95	9.40	0.54	5.87	5.89
4	2.4	0.54	2.51	14.04	7.25	15.80
5	7.79	3.25	8.44	10.12	0.91	10.16

Unit: pixel.

**Table 2:** Accuracy of fingertip recognition.

Number of fingertips	0	1	2	3	4	5	Average
Accuracy of recognition	98	99	98	97	100	98	98.3

Unit: %.

**Table 3:** Average processing times for arm pointing and finger counting gesture recognition.

	Arm pointing gesture recognition	Finger counting gesture recognition
Average processing time	6.1	0.5

Unit: ms.
